# 3D Janus plasmonic helical nanoapertures for polarization-encrypted data storage

**DOI:** 10.1038/s41377-019-0156-8

**Published:** 2019-05-15

**Authors:** Yang Chen, Xiaodong Yang, Jie Gao

**Affiliations:** Department of Mechanical and Aerospace Engineering, Missouri University of Science and Technology, Rolla, MO 65409 USA

**Keywords:** Metamaterials, Photonic devices

## Abstract

Helical structures have attracted considerable attention due to their inherent optical chirality. Here, we report a unique type of 3D Janus plasmonic helical nanoaperture with direction-controlled polarization sensitivity, which is simply fabricated via the one-step grayscale focused ion beam milling method. Circular dichroism in transmission of as large as 0.72 is experimentally realized in the forward direction due to the spin-dependent mode coupling process inside the helical nanoaperture. However, in the backward direction, the nanoaperture acquires giant linear dichroism in transmission of up to 0.87. By encoding the Janus metasurface with the two nanoaperture enantiomers having specified rotation angles, direction-controlled polarization-encrypted data storage is demonstrated for the first time, where a binary quick-response code image is displayed in the forward direction under the circularly polarized incidence of a specified handedness, while a distinct grayscale image is revealed in the backward direction under linearly polarized illumination with a specified azimuthal angle. We envision that the proposed Janus helical nanoapertures will provide an appealing platform for a variety of applications, which will range from multifunctional polarization control, enantiomer sensing, data encryption and decryption to optical information processing.

## Data storage: Helical holes for polarized encryption

Tiny helical apertures etched in gold could act as unit cells for data storage technologies based on the manipulation of polarized light. Helical nanostructures are suited to storing binary data because they exist in two forms, left- and right-handed helixes. Xiaodong Yang, Jie Gao and Yang Chen at Missouri University of Science and Technology used ion beam milling to carve arrays of helter-skelter-shaped helical ‘nanoapertures’ in 180-nanometer-thick gold films. Their samples allow selective transmission of certain types of polarized light, while blocking others. Moreover, this polarization sensitivity depends on the direction of the incoming light, meaning that when light is directed in one direction the array produces binary images such as QR codes, while in the other direction it can reproduce grayscale photographs. This opens exciting possibilities for data encryption and optical information processing.

## Introduction

Chirality, as first defined by Lord Kelvin, describes any geometrical figure or group of points whose mirror image cannot be brought to coincide with itself^1^. It is a ubiquitous property for biological objects, which range from small biomolecules, such as amino acids and nucleotides, to biological macromolecules, such as proteins and nucleic acids, and even to our hands and feet^2^. Although the right-handed and left-handed versions of a molecule, which are referred to as its two enantiomers, can have the same chemical and physical properties, they may possess entirely different biological functions; this promises important applications in flavor chemistry^3^, disease diagnosis^4^, and drug development^5,6^. Chiroptical analysis via circular dichroism (CD) spectroscopy is typically utilized to distinguish the two enantiomers^7,8^. However, chiroptical effects are extremely weak in natural materials. To overcome this problem, chiral plasmonic structures have been used to significantly boost the CD signals of chiral molecules. In addition to enantiomer sensing, chiral structures have been widely applied in miniature polarizers^9–11^, nonlinear optics^12–14^, and spin-controlled optical devices^15–18^.

Among the large family of chiral structures, helical nanostructures are particularly important since the electric field vector of circularly polarized light (CPL) also follows a helical trajectory^19,20^. Strong light–matter interaction is expected when the handedness of helical nanostructures matches that of CPL. However, the fabrication of helical nanostructures remains challenging. A three-dimensional (3D) plasmonic helix can be produced via two-photon direct laser writing followed by an electroplating step^9^; however, the micrometer-scale spatial resolution limits its application in the visible and near-infrared spectrum. Focused electron/ion beam-induced deposition can scale down the dimension of the helix structure to the nanoscale^21,22^; but the method suffers from low fabrication speed and, thus, cannot be used for large-scale production. The 3D nanocrescent, which is a simplified version of the helix, can be fabricated via the glancing angle deposition method^23,24^; however, the fabrication process is complicated and the CD signals are relatively low. In addition to adopting single structures with helical geometries, a twisted stack of achiral structures can also be organized via the aligned lithography technique to exhibit optical chirality^10,25,26^. However, lithography facilities with high-resolution alignment and delicate operations are required. In addition, the planar Archimedean spiral is proposed, of which the fabrication is less difficult^27,28^; however, it is not truly chiral and, hence, suffers from weak chiroptical responses. The fabrication of plasmonic helical nanostructures with giant CD signals in a fast and convenient way remains urgently necessary.

In this work, we propose and experimentally demonstrate a 3D Janus plasmonic helical nanoaperture as a new type of helical nanostructure. Despite possessing a truly chiral 3D geometry, the proposed helical nanoaperture can be easily fabricated via the one-step grayscale focused ion beam (FIB) milling method. In contrast to other types of helical nanostructures, the helical nanoaperture exhibits direction-controlled Janus polarization sensitivity. CD in transmission of as large as 0.72 is experimentally realized in the forward direction by exploiting the spin-dependent mode coupling process inside the nanoaperture. If the nanoaperture is illuminated in the backward direction, giant linear dichroism in transmission of up to 0.87 is acquired with high selectivity for the azimuthal angle of linearly polarized light. By encoding the specified rotation angles of the two nanoaperture enantiomers into the Janus metasurface, a direction-controlled polarization-encrypted data storage device is experimentally realized, which displays a binary quick response (QR) code image in the forward direction under circularly polarized incidence of a specified handedness, while showing another grayscale image in the backward direction under linearly polarized incidence of a specified azimuthal angle. Our work paves the way toward integrated photonic devices^29,30^, advanced enantiomer sensing^31,32^, data encryption and decryption^33,34^, and optical information processing^35,36^.

## Results

**Fig. 1:**
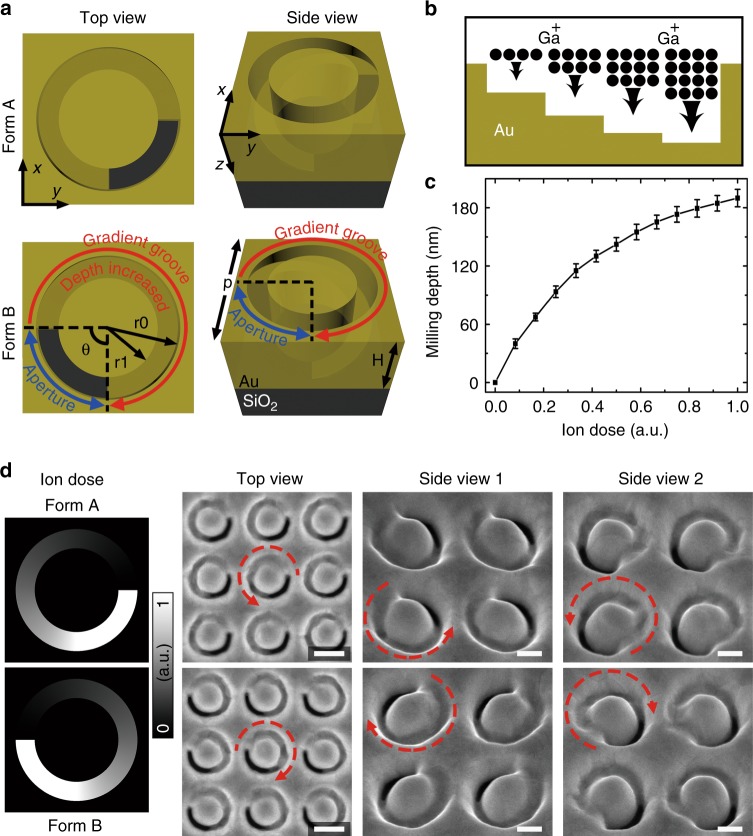
Design and fabrication of 3D Janus plasmonic helical nanoapertures. **a** A schematic diagram of the 3D Janus plasmonic helical nanoaperture in two enantiomeric forms: Form A and Form B. The geometric parameters are *p* = 380 nm, *r*0 = 160 nm, *r*1 = 110 nm, *θ* = 90°, and *H* = 180 nm. For Form B in the bottom row, the depth of the gradient groove part increases along the red arrow, while the aperture part is indicated by the blue double-arrow. **b** An illustration of the grayscale focused ion beam milling method. **c** The experimentally obtained milling depth as a function of the applied ion dose. **d** Normalized ion dose distributions and SEM images of the fabricated 3D helical nanoapertures. The side-view images are captured with a visual angle of 52° to the surface normal. The red dashed arrows indicate the direction along which the groove depth increases. The scale bars are 200, 100, and 100 nm from left to right

As illustrated in Fig. 1a, the 3D Janus plasmonic helical nanoaperture is etched in a single optically thick gold film and it is composed of an arc-shaped aperture and an arc-shaped gradient groove that are connected end to end with each other. In contrast to the conventional aperture or groove structure with a uniform depth, the depth of the gradient groove is gradually increased from zero (not etched) to the gold-film thickness *H* (totally etched through), which is critical for the proposed helical nanoaperture to acquire true 3D chirality. Depending on whether the depth of the gradient groove is increased counterclockwise or clockwise, the chiral helical nanoapertures exist in two enantiomeric forms, namely, Form A and Form B, which are mirror-symmetric with each other.

Despite its 3D geometry, the helical nanoaperture can be easily fabricated in only one step by using the grayscale FIB milling method (Fig. 1b), where a higher Ga^+^ ion dose is applied to locally obtain a larger milling depth. Due to the substantial redeposition effect inside the nanoaperture with a high depth-to-width ratio, the milling depth per unit of ion dose is gradually reduced as the groove depth is increased (Fig. 1c)^37^. This effect should be considered and compensated when editing the grayscale milling patterns (Fig. 1d). By delicately adjusting the focus and astigmatism of the ion beam, 3D helical nanoaperture arrays with satisfactory uniformity are produced, as shown in Fig. 1d. To further improve the aperture uniformity, we can use the He^+^-FIB system with a higher milling resolution and the gold film with a finer grain size and higher film density.

**Fig. 2:**
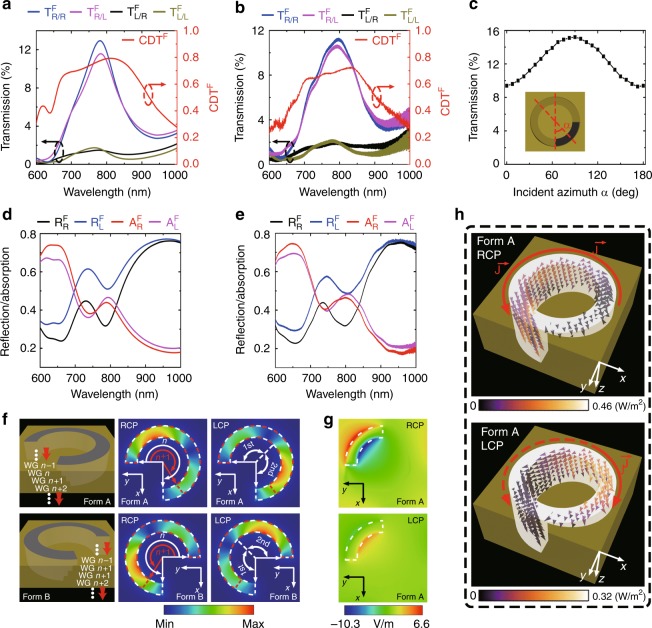
Optical properties of the 3D Janus plasmonic helical nanoaperture in the forward direction. **a** Simulated and **b** measured transmission spectra of the helical nanoaperture array in Form A for various incident/output handedness combinations in the forward direction, together with the corresponding CDT^*F*^ spectra. **c** The measured transmission intensity as a function of the azimuthal angle *α* of the LP incident light at 830 nm. **d** Simulated and **e** measured reflection spectra ($R_R^F$ and $R_L^F$) and absorption spectra ($A_R^F$ and $A_L^F$) under RCP and LCP incidence in the forward direction. **f** Illustrations of the spin-dependent mode coupling processes inside the 3D helical nanoapertures in Form A and Form B, which can be considered as series of cascaded waveguide segments (*WG n*−1, *WG n*, *WG n* + 1,…). Circularly dichroic mode distributions are presented inside the waveguide segment *WG n* at 812 nm. **g** Electric field distributions 6 nm above the exit plane of the helical nanoaperture in Form A under RCP and LCP incidence at 812 nm. **h** Optical power flow distributions inside the helical nanoaperture in Form A under RCP and LCP incidence at 812 nm

First, the chiroptical properties of 3D plasmonic helical nanoapertures are studied in the forward direction, when CPL is illuminated onto the gold surface and transmitted out from the silica substrate. Enantiomers in Form A are arranged in a periodic array. The simulation results in Fig. 2a demonstrate a significant transmission resonance at 783 nm for right-handed circularly polarized (RCP) incidence, while the transmission is strongly suppressed for left-handed circularly polarized (LCP) incidence at this wavelength. We define CD in transmission in the forward direction, which is denoted as CDT^*F*^, as

1 $${\mathrm{CDT}}_{}^F = \frac{{(T_{R/R}^F + T_{R/L}^F) - (T_{L/R}^F + T_{L/L}^F)}}{{(T_{R/R}^F + T_{R/L}^F) + (T_{L/R}^F + T_{L/L}^F)}}$$

where $T_{R/L}^F$ refers to the intensity of the LCP transmission component under the forward RCP incidence. Accordingly, a broadband *CDT*^*F*^ resonance is observed with a value that exceeds 0.5 from 654 to 922 nm. The peak value of 0.79 is attained at 812 nm. In the experiment, the CDT^*F*^ resonance is measured to be larger than 0.5 from 680 to 900 nm and the maximum value of 0.72 is attained at 830 nm (Fig. 2b). The simulation and experimental results approximately coincide with each other and the discrepancies are mainly due to the fabrication tolerance of the FIB system, the quality of the gold film and the imperfections of the optical components in the experimental setup. The polarization state of the transmitted light under RCP incidence is analyzed in Supplementary Information S1. Moreover, due to the large CDT^*F*^ of the nanoaperture, the transmission intensity under linearly polarized (LP) incidence shows a relatively weak dependence on the azimuthal angle of the LP light (Fig. 2c), where the RCP component of the LP light is selectively transmitted and the LCP component is preferentially blocked.

For most helical structures, CD primarily originates from the circularly dichroic ohmic loss distributions inside the structure; hence, a structure thickness that is comparable to the wavelength is required for accumulating an absorption difference and producing large CD signals^10,22^, which is unfavorable for device fabrication and integration. However, for the proposed helical nanoaperture, high CD in transmission is realized over a thickness of less than *λ*/4 in the forward direction, thereby indicating a distinct physical origin. According to the reflection and absorption spectra that are shown in Fig. 2d, e, the large transmission difference of the helical nanoaperture is mainly attributed to the circularly dichroic response of reflection rather than absorption. However, the direct reflection from the nanoaperture entrance plane with the mirror-symmetric arc-shaped contour is the same for both RCP and LCP incidence. It follows that the spin-dependent mode coupling process occurs when the coupled circularly polarized wave propagates inside the nanoaperture. The 3D helical nanoaperture can be modeled as a series of cascaded arc-shaped waveguide segments (Fig. 2f), whose flare angles are gradually reduced from almost 360° at the entrance plane to 90° at the exit plane. The flare angle reduction directions are opposite for Form A and Form B. At the interface of two connected waveguide segments, which we denote as *WG n* and *WG n* + 1, the coupled propagation wave inside segment *WG n* is reflected back or transmitted into segment *WG n* *+* *1* depending on the mode coupling process, which is further determined by the field overlap conditions between the two segments. For arc-shaped waveguide segment *WG n*, circularly dichroic mode distributions are demonstrated, which are primarily localized in the first half for the RCP case and in the second half for the LCP case (Fig. 2f). Although we only show the case with a flare angle of 270°, such spin-dependent mode distributions are universal for arbitrary flare angles from 90° to almost (but not) 360°, as discussed in Supplementary Information S2. If the 3D helical nanoaperture in Form A is considered, segment *WG n* + 1 has superior field overlap with segment *WG n* for the RCP case compared to the LCP case, thereby leading to more optical power being transmitted into segment *WG n* + 1 via the mode coupling process (Supplementary Information S3). Hence, the reflection of the RCP mode is weaker at the interface. As the coupled wave propagates from the entrance plane of the nanoaperture to the exit plane, the mode coupling difference is accumulated continually, thereby resulting in a much larger net transmission and a smaller net reflection for RCP incidence compared to those for LCP incidence. We also plot the electric field distributions at the exit plane of Form A; the electric field is much stronger for the RCP case (Fig. 2g). Thus, the optical signal into the transmission side is significantly enhanced compared to the LCP case. For Form B, segment *WG n* + 1 has a superior field overlap with the LCP mode of segment *WG n*, thereby generating the opposite chiroptical response (Fig. 2f).

The optical power flow distributions in Fig. 2h further demonstrate the importance of the gradient groove structure for the 3D helical nanoaperture to acquire optical chirality. If the handedness of CPL matches that of the gradient groove, such as RCP light for Form A or LCP light for Form B, the incoming optical power is collected and guided into the aperture area along the gradient groove to produce a strong transmission. Otherwise, the accepted optical power is directed away from the aperture area, thereby resulting in a weak transmission. Compared to an arc-shaped nanoaperture that lacks the gradient groove structure, the 3D helical nanoaperture in Form A can produce a stronger transmission under RCP incidence, but a weaker transmission under LCP incidence (see Supplementary Information S4). While the gradient groove part of the 3D helical nanoaperture acts as a spin-dependent optical power director, the arc-shaped aperture part functions as a LP emitter. The optical power that is accepted by the gradient groove is coupled to the fundamental dipole mode of the arc-shaped aperture, which generates LP emission that is perpendicular to the arc-shaped aperture (Fig. 2g). Consequently, the transmitted RCP and LCP components, namely, $T_{R/R}^F$ and $T_{R/L}^F$, have almost equal intensity in the forward direction (Fig. 2a, b).

Next, the optical properties of 3D plasmonic helical nanoapertures in the backward direction are investigated, where light is illuminated onto the silica substrate and transmitted out from the gold surface. Three-dimensional chiral structures such as the 3D helix and the twisted stack maintain their sense of twist if observed from the opposite side. However, 2D chiral structures such as the Archimedean spiral will exhibit a reversed sense of rotation if illuminated in the opposite direction, thereby producing an asymmetric transmission effect^38,39^. Here, the forward transmission properties of the 3D helical nanoaperture can be expressed using circular Jones transmission matrix $T_{circ}^{F}$ as:

2 $$T_{circ}^{F} = \left[ {\begin{array}{*{20}{c}} {t_{R/R}^F} & {t_{L/R}^F} \\ {t_{R/L}^F} & {t_{L/L}^F} \end{array}} \right]$$

where $t_{R/L}^F$ denotes the complex transmission coefficient of the LCP component under forward RCP illumination. Based on the Lorentz reciprocity theorem, its backward transmission properties can be characterized as:

3 $$T_{circ}^{B} = \left[ {\begin{array}{*{20}{c}} {t_{R/R}^B} & {t_{L/R}^B} \\ {t_{R/L}^B} & {t_{L/L}^B} \end{array}} \right] = \left[ {\begin{array}{*{20}{c}} {t_{R/R}^F} & { - t_{R/L}^F} \\ { - t_{L/R}^F} & {t_{L/L}^F} \end{array}} \right]$$

**Fig. 3:**
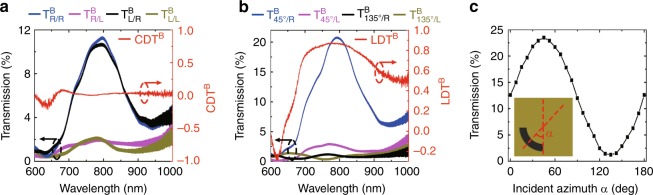
Optical properties of the 3D Janus plasmonic helical nanoaperture in the backward direction. **a** Measured transmission spectra of the helical nanoaperture array in Form A for various incident/output handedness combinations in the backward direction, together with the corresponding CDT^*B*^ spectrum. **b** Measured transmission spectra for various polarization combinations of LP-incident/CP-output in the backward direction, together with the corresponding LDT^*B*^ spectra. **c** The measured transmission intensity as a function of the azimuthal angle *α* of the LP incident light at 800 nm

The two diagonal elements, which represent the co-polarized transmission, remain unchanged, while the two off-diagonal elements, which represent the cross-polarized transmission, are reversed in sign and exchanged^40^, which is experimentally verified in Fig. 3a. For the helical nanoaperture in Form A, RCP incidence and LCP incidence are transmitted with similar intensities and have the same right-handed circular polarization. Backward CD in transmission CDT^*B*^ is calculated to be almost zero from 650 to 1000 nm. Although the proposed nanoaperture loses its spin sensitivity for the oppositely illuminated light, it acquires high-transmission selectivity for the azimuthal angle of LP light. As shown in Fig. 3b, the LP light with an azimuthal angle *α* of 45° is resonantly transmitted, while the LP light in the orthogonal direction (*α* = 135°) is effectively blocked. Backward linear dichroism in transmission LDT^*B*^ is defined as

4 $${\mathrm{LDT}}_{}^B = \frac{{(T_{45^\circ /R}^B + T_{45^\circ /L}^B) - (T_{135^\circ /R}^B + T_{135^\circ /L}^B)}}{{(T_{45^\circ /R}^B + T_{45^\circ /L}^B) + (T_{135^\circ /R}^B + T_{135^\circ /L}^B)}}$$

which exceeds 0.75 from 700 nm to 870 nm. At the resonant wavelength of 800 nm, the transmission intensity is measured to approximately follow a cosine function with respect to the incident azimuthal angle *α* (Fig. 3c). The underlying mechanism can be explained as follows: the arc-shaped aperture part that faces the backward incoming wave acts as a LP receiver, where only the LP component that is perpendicular to the aperture is accepted. Since RCP and LCP incident light are of the same intensity as the LP component that is perpendicular to the aperture, no CD is exhibited in the backward direction. Furthermore, the accepted optical power is coupled to the RCP mode of the gradient groove structure, thereby giving rise to RCP transmission with almost the same intensity as $T_{R/R}^B$ and $T_{L/R}^B$ in the backward direction (Fig. 3a).

**Fig. 4:**
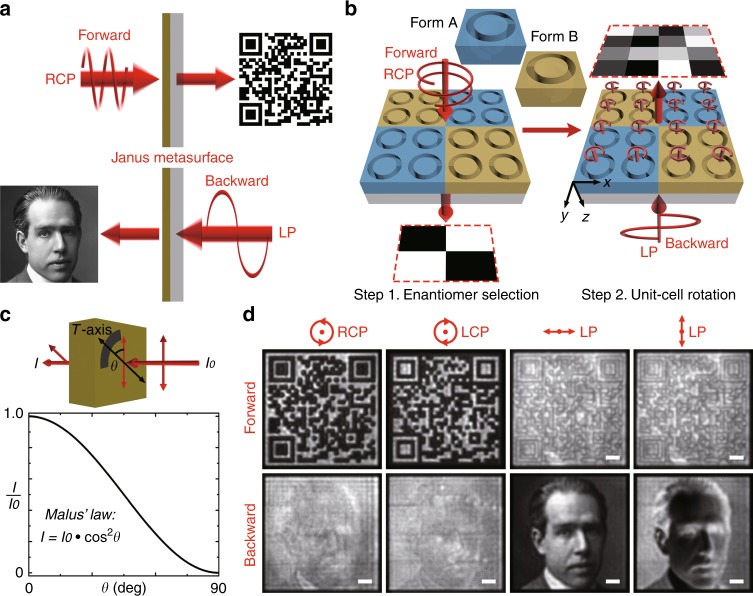
Direction-controlled polarization-encrypted data storage with the Janus metasurface. **a** A schematic diagram of the Janus metasurface for direction-controlled polarization-encrypted data storage. (Photograph used with permission: Niels Henrik David Bohr (1885–1962) Danish physicist. Quantum Theory. Nobel prize for physics 1922/Universal History Archive/UIG/Bridgeman Images.) **b** An illustration of the metasurface encoding process with the two nanoaperture enantiomers having specified rotation angles. **c** The normalized transmission intensity of LP light in the backward direction, which follows Malus’s law with respect to the angle *θ* between the incident polarization direction and the transmission axis of the 3D helical nanoaperture. **d** Captured transmission images of the Janus metasurface at 800 nm in both the forward and backward directions for various incident polarizations. Scale bar: 10 μm

Recently, other types of chiral nanostructures with high CD have been proposed, such as the folded split ring resonator (SRR)^41^ and the stepped nanoaperture^16^. However, the handedness of the circularly polarized incidence, if selectively transmitted, is maintained or flipped during the transmission process for the folded SRR or the stepped nanoaperture. As a result, if illuminated in the opposite direction, the chirality is either maintained or reversed according to the Lorentz reciprocity theorem. However, for the current 3D helical nanoaperture, the selective circularly polarized incidence is converted into LP output in the forward direction with large CD, whereas in the backward direction the CD disappears and, instead, giant linear dichroism is exhibited with circularly polarized output. The demonstrated 3D helical nanoaperture, which has extraordinary direction-dependent polarization sensitivity, provides an ideal platform for direction-controlled polarization-encrypted data storage applications. Its rounded shape is also beneficial for unit-cell rotation and near-field coupling weakening (see Supplementary Information S5). As illustrated in Fig. 4a, the designed Janus metasurface can display a binary QR code image in the forward direction under CPL illumination of a specified handedness, while it will display a grayscale image in the backward direction under LP incidence with a suitable polarization direction. To encode the Janus metasurface, the first step is to determine whether enantiomer A or B is selected for each unit cell so that an alternative color of white or black is locally defined for constructing the binary QR code in the forward direction under RCP illumination (Fig. 4b). In the second step, each unit cell is independently rotated at a specified angle to generate a grayscale pixel in the backward direction under *x*-polarized incidence. The nanoaperture rotation angle is determined based on Malus’s law, according to which the local transmission intensity is proportional to the square of the cosine of the angle *θ* between the incident polarization direction and the transmission axis of the unit-cell nanoaperture (Fig. 4c). The rotation of the nanoaperture does not influence its forward transmission properties under CPL and the selection of enantiomers has little impact on its backward transmission characteristics under LP light. As a result, the information of the forward binary image and the backward grayscale image can be encoded into the same Janus metasurface independently without mutual disturbance.

Transmission images are recorded with a camera at a wavelength of 800 nm (Fig. 4d). Although the CDT^*F*^ value at this wavelength is slightly smaller than the maximum value, the LDT^*B*^ value and the transmission efficiencies for both directions are dramatically increased. In the forward direction, only when RCP light is illuminated can a QR code image be decrypted and revealed, which can directly connect to the Wikipedia web link of the famous physicist Niels Bohr upon being scanned by a portable QR code scanner. When the incident handedness is switched to LCP, a complementary binary image is captured, which cannot be recognized by the QR code scanner. For the case of LP incident light, the contrast between the black and white squares in the QR code image disappears. Once the illumination direction has been inverted, no clear image can be distinguished from the nearly homogeneous background under CPL incidence. Instead, an *x*-polarized light beam can decode the metasurface to show a grayscale portrait image of Niels Bohr with high fidelity. If the incident polarization is changed to *y*-polarization, a complementary grayscale image is formed according to Malus’s law, where the original bright pixels become dark and vice versa. Therefore, by using the 3D Janus plasmonic helical nanoapertures as unit cells, direction-controlled polarization-encrypted data storage has been experimentally realized for the first time. Two images are simultaneously stored in a single Janus metasurface, which can be separately read out in the forward and backward directions only if the incident polarization that acts as the decryption key is applied.

**Fig. 5:**
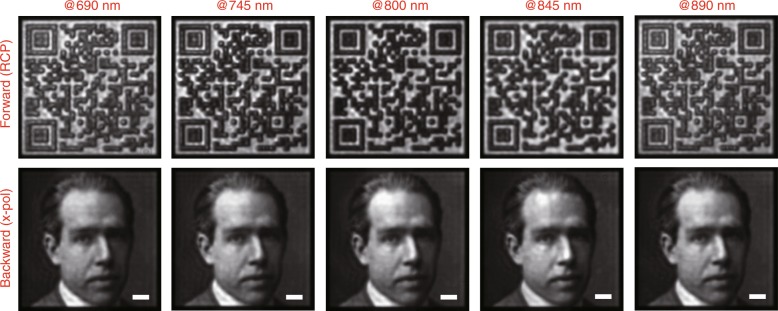
Broadband performance of the Janus metasurface. The images are captured under proper illumination conditions of direction and polarization at 690, 745, 800, 845, and 890 nm. Scale bar: 10 μm

Due to the resonant properties of 3D plasmonic helical nanoapertures, there is a working bandwidth for the encoded Janus metasurface, which is mainly determined by the CDT^*F*^ spectrum of the nanoaperture. Away from the resonant wavelength, the CDT^*F*^ value is reduced, thereby resulting in deteriorated contrast between the white and black squares of the QR code image. The QR code image can still be distinguished by a QR code scanner at 690 and 890 nm, where the measured CDT^*F*^ value is approximately 0.56 (Fig. 5). Further reduction of the *CDT*^*F*^ value to 0.48 at 670 nm and 910 nm can cause the recognition success rate to decrease sharply to zero. In the wavelength range from 690 to 890 nm, the LDT^*B*^ value well exceeds 0.70 and the grayscale images that are captured in the backward direction are always of high quality (Fig. 5). Consequently, the working bandwidth for the currently demonstrated Janus metasurface is approximately 200 nm and ranges from 690 to 890 nm.

## Discussion

A new type of 3D Janus plasmonic helical nanoaperture with direction-switched polarization sensitivity has been introduced. This nanoaperture can be simply fabricated via the one-step grayscale FIB milling method. Due to the spin-dependent mode coupling process inside the helical nanoaperture, CD in transmission of as large as 0.72 is generated in the forward direction. When the helical nanoaperture is illuminated from the backward direction, it exhibits no transmission selectivity for the handedness of circularly polarized light; however, it acquires giant linear dichroism in transmission of as high as 0.88. The unique optical properties of the 3D helical nanoaperture enable direction-controlled polarization-encrypted data storage that is based on the encoded Janus metasurface, where two images, namely, a binary image and a grayscale image, can be separately displayed in the forward and backward directions under an incident polarization. Our demonstrated Janus helical nanoapertures will find additional applications in multifunctional polarizers, high-resolution display, chiral sensing, data encryption and decryption, and optical information processing.

## Materials and methods

### Numerical modeling

Numerical simulations are conducted using a commercial finite-element solver (COMSOL Multiphysics). The refractive index of silica is set to 1.45 and the permittivity of gold is extracted from spectroscopic ellipsometry data that are fitted with a general oscillator model. The unit-cell structure is surrounded by periodic boundary conditions in the horizontal direction, while it is truncated by perfectly matched layers that are enclosed by a scattering boundary in the vertical direction to prevent reflection (Fig. S4a). Normal incidence of a plane wave under a specified polarization is introduced.

### Sample fabrication

A FIB system (Helios Nanolab 600, 30 kV, 9.7 pA) is utilized to fabricate 3D Janus plasmonic helical nanoapertures in a 180-nm-thick gold film, which is deposited on a silica substrate via electron beam evaporation. The influence of Ga^+^ ion accumulation is not considerable in our experiments due to the low ion current and use of conductive copper tape. In the metasurface design, the QR code image consists of a 31 × 31 array of squares in black or white color. Each square is further composed of a 7 × 7 array of nanoaperture unit cells. Thus, the metasurface possesses an overall size of 82 μm × 82 μm. The fabrication times for a single unit cell and the total metasurface are approximately 0.2 s and 2.6 h, respectively. SEM images of the metasurface device are shown in Fig. S8.

### Optical characterization

For transmission spectrum measurements of the 3D helical nanoapertures, a collimated broadband light beam from a tungsten halogen light source (Thorlabs) is passed through a linear polarizer and a quarter-wave plate and focused normally onto the sample using a 20× objective. The transmitted light is collected by another 20× objective, directed through a quarter-wave plate that is cascaded by a linear polarizer and received by a spectrometer (Horiba, iHR 550) for circular polarization analysis (Fig. S7). If LP incidence is required, the quarter-wave plate before the sample is replaced by a half-wave plate. To capture the transmission images of the Janus metasurface in the forward direction, a collimated laser beam that is emitted from a tuneable Ti:Sapphire oscillator (Chameleon Ultra, Coherent) is directed through a linear polarizer and a quarter-wave plate and focused on the sample using a lens (Fig. S7). The transmission image is collected and magnified by a 20× objective and captured by an infrared-CCD camera. For optical imaging in the backward direction, the quarter-wave plate is replaced by a half-wave plate to rotate the polarization direction of the linearly polarized beam. The captured QR code image is displayed on a computer screen and directly recognized by ordinary QR code scanner software that is installed in an Apple iPhone 7.

## Supplementary information

SUPPLEMENTAL INFORMATION for 3D Janus plasmonic helical nanoapertures for polarization-encrypted data storage
