# Supplementary material for: 3D Janus plasmonic helical nanoapertures for polarization-encrypted data storage
Source: Light Sci Appl. 2019 May 15;8:45. doi: 10.1038/s41377-019-0156-8 (PMC6517422; doi:10.1038/s41377-019-0156-8)
Supplement: Supplementary file 1 — SUPPLEMENTAL INFORMATION for 3D Janus plasmonic helical nanoapertures for polarization-encrypted data storage [file 41377_2019_156_MOESM1_ESM.docx]

Supplementary Information for

3D Janus plasmonic helical nanoapertures for polarization-encrypted data storage

*Yang Chen, Xiaodong Yang*, and Jie Gao**

Department of Mechanical and Aerospace Engineering, Missouri University of Science and Technology, Rolla, MO 65409, USA

*E-mail: (X. Y.) [yangxia@mst.edu](mailto:yangxia@mst.edu), (J. G.) [gaojie@mst.edu](mailto:gaojie@mst.edu).

**S1. Output polarization state in the forward direction under RCP incidence**


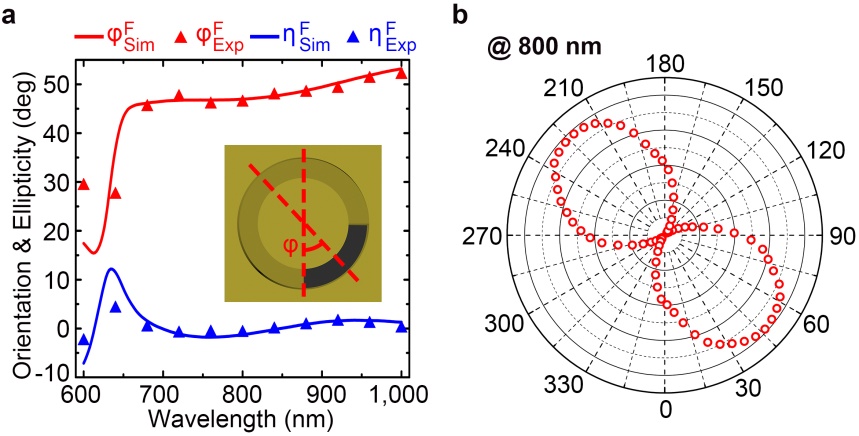


**Fig. S1. (a)** Output polarization orientation angle *φ* and ellipticity angle *η* of the 3D plasmonic helical nanoaperture array under RCP incidence in the forward direction obtained from simulation and experiment. (b) Polar diagram of the output polarization measured at 800 nm.

As discussed in the manuscript, 3D plasmonic helical nanoapertures are demonstrated to possess high circular dichroism in transmission in the forward direction. The RCP light is resonantly transmitted by the enantiomer A, while the LCP light is effectively blocked. The polarization state of the transmitted light for the RCP incident case is analyzed here. Over a broad spectrum from 680 nm to 1000 nm, although the orientation angle *φ* is slightly increased, it remains nearly orthogonal to the arc-shaped aperture. The ellipticity angle *η* is measured to be below 1.5° indicating good polarization linearity. The linearly polarized transmission can be attributed to the dipole emission of the arc-shaped aperture at the exit plane, which is clearly revealed by the electric field distributions in Fig. 2g. Polar diagram at 800 nm further shows a typical dipole emission pattern (Fig. S1b).

**S2. Spin-dependent mode distributions for the arc-shaped waveguide**


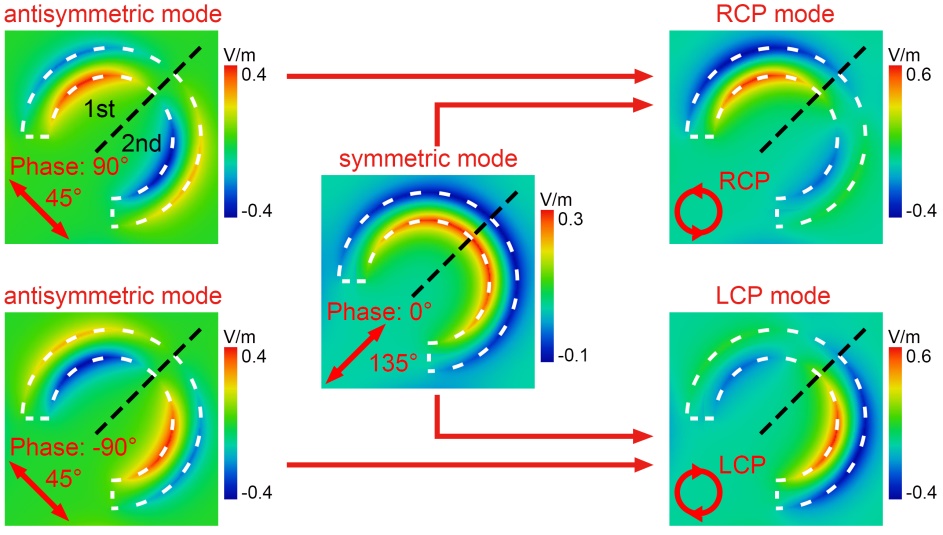


**Fig. S2.** Electric field Ez distributions of the arc-shaped waveguide under 45°*-*polarized and 135°-polarized incidence with certain phase delay at 812 nm, and the resulting Ez distributions under RCP and LCP illumination.

The spin-dependent mode distributions inside the arc-shaped waveguide are originated from the different interference between the antisymmetric mode and symmetric mode of the waveguide. As shown in Fig. S2, when a LP light perpendicular to the symmetry axis is illuminated, the antisymmetric plasmonic mode of the arc-shaped waveguide is excited, where two electric dipoles with opposite phases are induced at the two halves. But if a LP light parallel to the symmetry axis is illuminated, the symmetric plasmonic mode is excited with a single electric dipole induced around the center of the waveguide. Furthermore, when circularly polarization excitation is utilized, the two plasmonic modes are simultaneously excited with a relative phase delay of 90° or -90° and interfere with each other to generate the RCP and LCP modes. For the RCP case, the interference is locally constructive at the first half and destructive at the second half, resulting in the electric field enhanced at the first half and weakened at the second half. The situation is reversed for the LCP case. Chiral mode distributions are thus generated inside the arc-shaped waveguide.


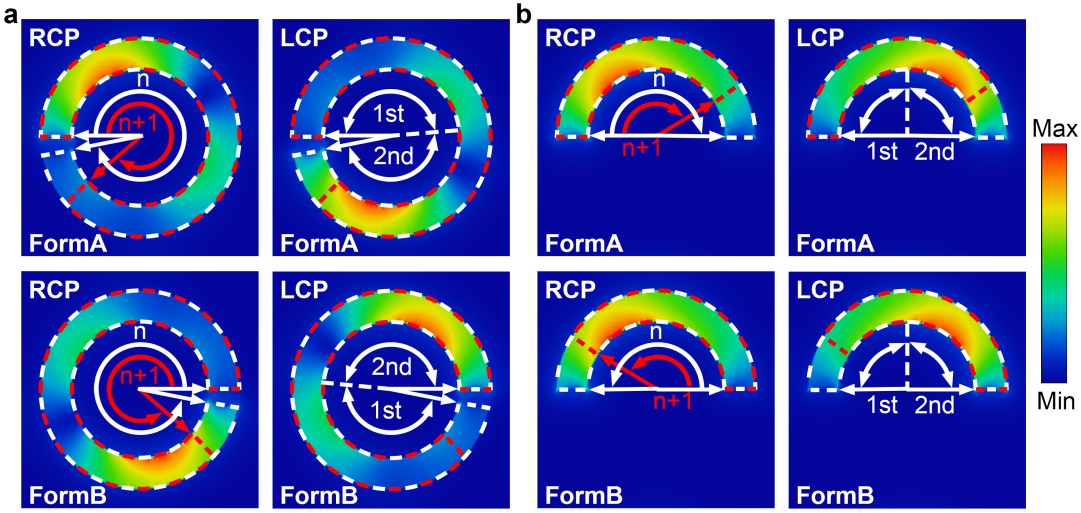


**Fig. S3.** Electric field distributions inside the waveguide segment *WG n* at 812 nm with a flare angle of **(a)** 350° and **(b)** 180°.

In fact, the spin-dependent mode distributions are universal for the arc-shaped waveguide with an arbitrary flare angle from 90° to almost (but not) 360°. Fig. S3 shows the electric field distributions for the flare angle of 350° and 180 °. It is observed that the mode field is mainly localized at the first half for the RCP case but at the second half for the LCP case, which is similar to the case of 270°.

**S3. Circularly dichroic mode coupling difference at the interface**

**
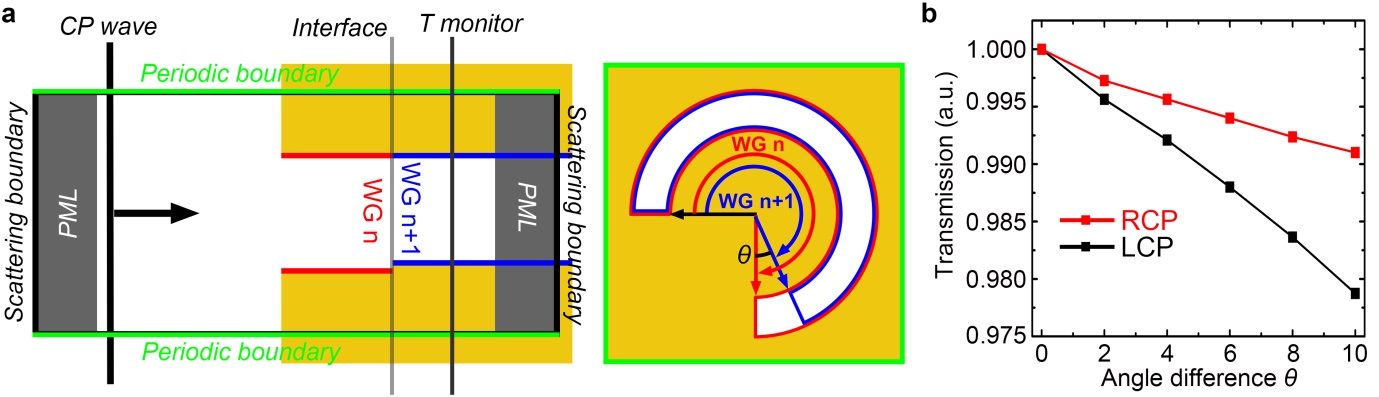
**

**Fig. S4. (a)** Illustration of the simulation setup. **(b)** Normalized transmission intensity for the RCP and LCP incident case obtained at the *T monitor* as a function of the angle difference *θ*.

As described in the manuscript, spin-dependent mode coupling process occurs at the interface of two waveguide segments *WG n* and *WG n+1*. Better field overlap between the two segments leads to more optical power transmitted from *WG n* to *WG n+1*. Here, we are to verify the conclusion based on numerical simulations. As shown in Fig. S4a, circularly polarized plane wave (RCP or LCP) is illuminated onto the arc-shaped waveguide *WG n* followed by another arc-shaped waveguide *WG n+1*, whose flare angle is smaller than *WG n*. A series of simulations are conducted with the flare angle difference *θ* gradually increased from 0° to 10°, while the corresponding transmission intensity is monitored after the interface for both RCP and LCP incidence conditions. As depicted in Fig. S4b, benefitting from the better field overlap at the interface, the transmission intensity of the RCP case is stronger compared to the LCP case at the *T monitor*, indicating more optical power coupled into the waveguide *WG n+1* for the RCP case. Such a coupling difference is enlarged with an increased *θ*, which is attributed to the growing field mismatch between the two waveguides under LCP excitation.

**S4. Comparison of the 3D helical nanoaperture and the arc-shaped nanoaperture**


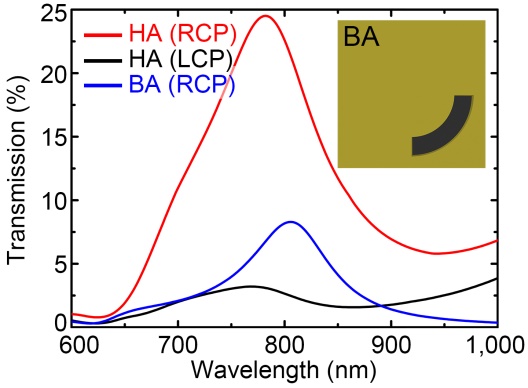


**Fig. S5.** Simulated transmission spectra of the arc-shaped nanoaperture (BA) as shown in the inset and the 3D helical nanoaperture (HA) in the forward direction.

For the ordinary arc-shaped nanoaperture without the gradient groove structure, a transmission resonance is observed at 806 nm (Fig. S5), corresponding to the fundamental dipole mode. No chirality is expected for its mirror-symmetric shape. If the gradient groove structure is introduced, it functions as a spin-dependent optical power director. The incident RCP wave is collected and focused along the gradient groove into the aperture area to generate an enhanced transmission, while the coupled LCP waves are directed away from the aperture area, resulting in a suppressed transmission (Fig. S5).

**S5. Near-field coupling between unit cells**

**
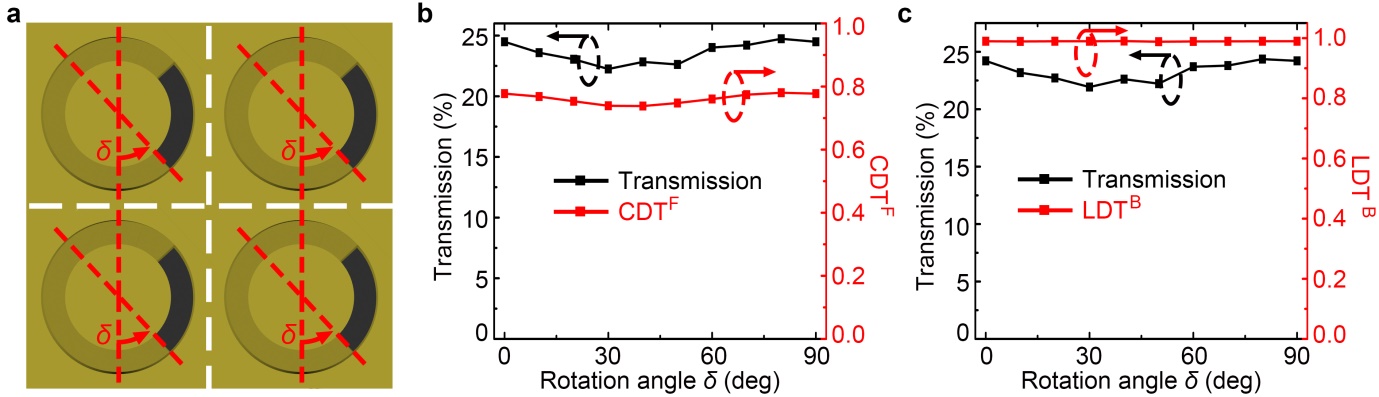
**

**Fig. S6. (a)** Schematic of the uniform array of 3D plasmonic helical nanoapertures in Form A with a rotation angle of *δ*. **(b)** Transmission intensity under RCP incidence and the corresponding CDT amplitude in the forward direction as a function of the rotation angle *δ* at 830 nm. **(c)** Transmission intensity under LP incidence of 45° and the corresponding LDT amplitude in the backward direction as a function of the rotation angle *δ* at 800 nm.

The circular shape of the 3D helical nanoaperture is beneficial for avoiding the near-field coupling effect between unit cells in the metasurface applications, because no sharp corner is presented and the distance between adjacent unit cells is maintained. To quantitatively evaluate such effect, a uniform array of 3D plasmonic helical nanoapertures with varied rotation angles are simulated in the forward and backward directions (Fig. S6a). In the forward direction, the oscillation of the RCP transmission intensity and CDT amplitude is within 5% and 3% of the corresponding average values. Meanwhile, in the backward direction, the transmission intensity of the LP light of 45° and LDT amplitude is changed within 5% and 1% of the corresponding average values. As a conclusion, the near-field coupling effect between unit cells is effectively suppressed for our proposed helical nanoapertures.

**S6. Optical characterization setup**

**
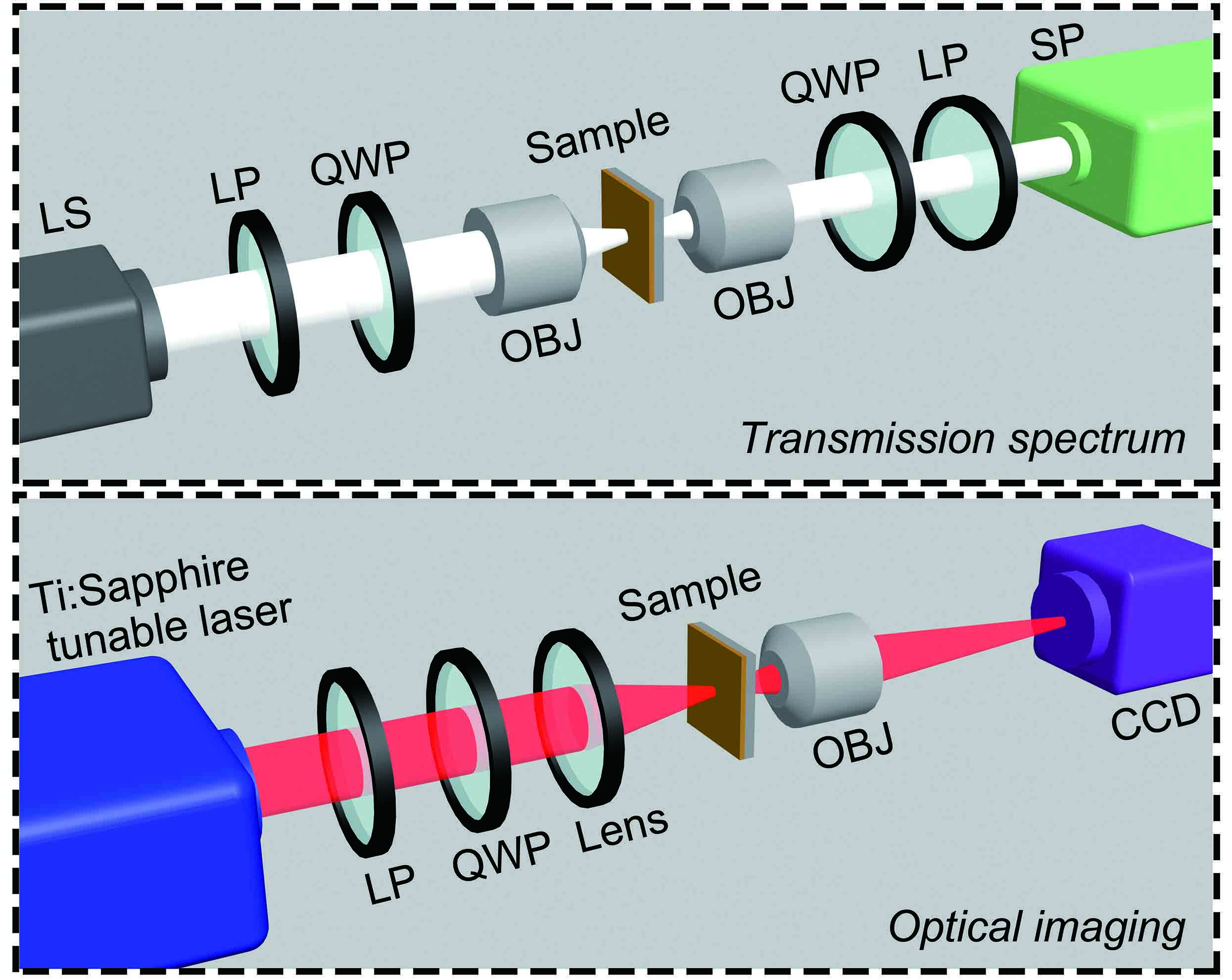
**

**Fig. S7.** Schematic illustration of the optical characterization setups. LS, W-Halogen white light source; SP, spectrometer; LP, linear polarizer; QWP, quarter-wave plate; OBJ, objective.

**S7. SEM images of the metasurface device**

**
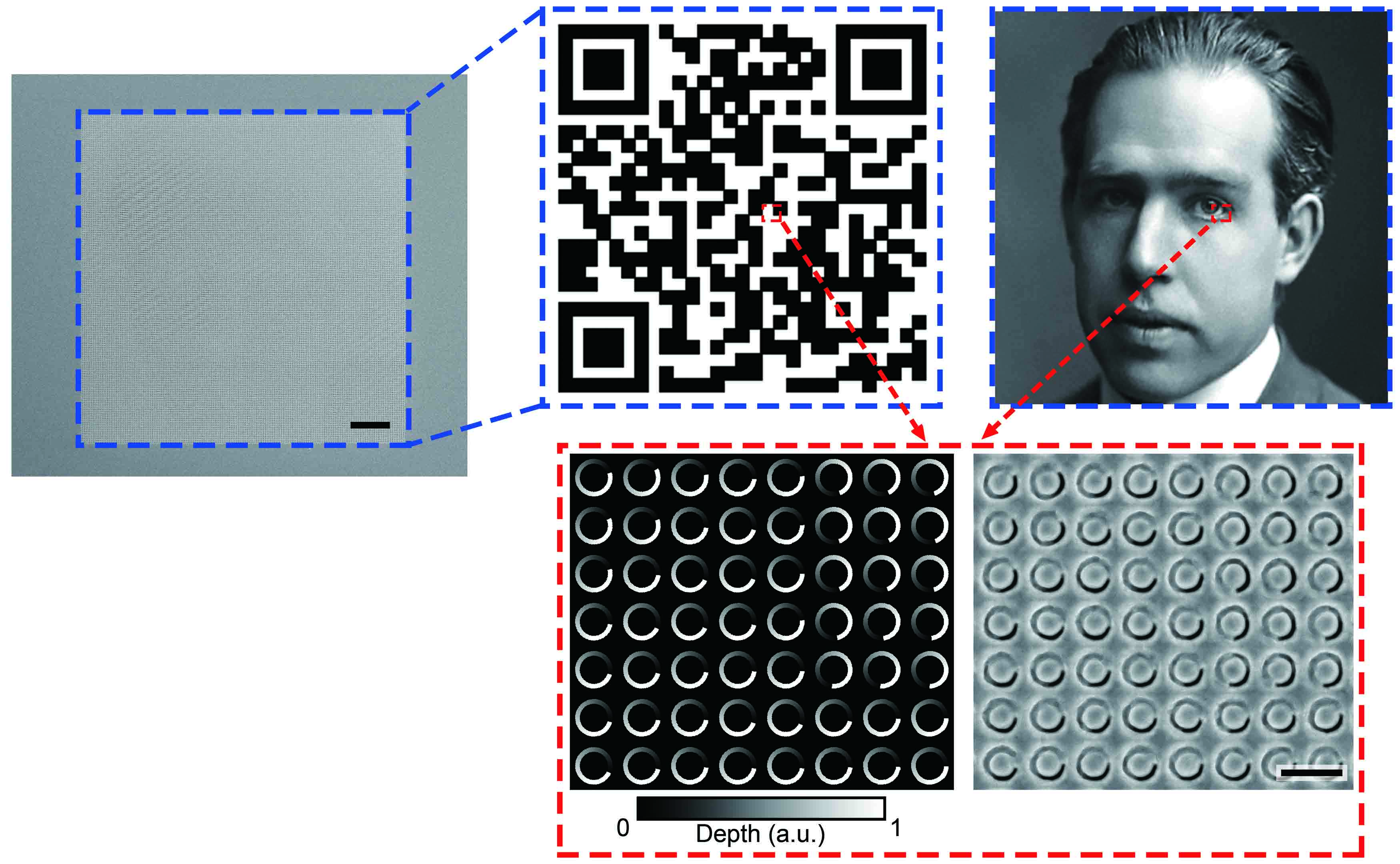
**

**Fig. S8.** Overall (upper left, scale bar: 10 μm) and partial (lower right, scale bar: 500 nm) SEM images of the metasurface device.
